# The evolving role of targeted radioligand therapy in small cell and non-small cell lung cancer: a systematic review

**DOI:** 10.37349/etat.2026.1002368

**Published:** 2026-04-27

**Authors:** Serin Moghrabi, Saad Ruzzeh, Kamal Al-Rabi, Ahmed Abdlkadir, Mohammed J. Al-Jaghbeer, Nouraldeen Alzorgan, Ula Al Rasheed, Mohammad Alqudah, Akram Al-Ibraheem

**Affiliations:** IRCCS Istituto Romagnolo per lo Studio dei Tumori (IRST) “Dino Amadori”, Italy; ^1^Department of Nuclear Medicine, King Hussein Cancer Center (KHCC), Amman 11941, Jordan; ^2^Medical Oncology and Hematology Department, King Hussein Cancer Center (KHCC), Amman 11941, Jordan; ^3^Pulmonary and Critical Care Department, King Hussein Cancer Center (KHCC), Amman 11941, Jordan; ^4^Faculty of Medicine, Hashemite University (HU), Zarqa 13133, Jordan; ^5^Division of Nuclear Medicine, Department of Radiology and Nuclear Medicine, University of Jordan, Amman 11942, Jordan

**Keywords:** SCLC, NSCLC, targeted radionuclide therapy, theranostics, PRRT

## Abstract

**Background:**

Targeted radioligand therapy (TRT) is an emerging theranostic modality in oncology. While well established in neuroendocrine and prostate cancers, its role in small cell lung cancer (SCLC) and non-small cell lung cancer (NSCLC) remains investigational. This systematic review summarizes current evidence evaluating TRT in lung cancer.

**Methods:**

A Preferred Reporting Items for Systematic reviews and Meta-Analyses (PRISMA)-guided systematic review of PubMed, Embase, and Scopus (2000–November 2025) was conducted. Original studies evaluating TRT in SCLC or NSCLC were included. Primary outcomes were tumor response, disease-control rate, and treatment-related toxicity. Secondary outcomes included progression-free survival, overall survival, and dosimetry. Risk of bias was assessed using the Risk Of Bias In Non-randomized Studies—of Interventions (ROBINS-I) tool.

**Results:**

From 2,453 records, 15 studies were included, reporting 358 lung cancer patients, of whom 105 received TRT. Disease-control rates reached up to 78% in mixed NSCLC/SCLC cohorts. In SCLC, somatostatin receptor-targeted peptide receptor radionuclide therapy demonstrated heterogeneous disease control (0–50%), with [^177^Lu]Lu-labeled agents showing more favorable outcomes than [^90^Y]Y-based therapy. The most favorable outcomes were a median progression-free survival of 11.9 months and an overall survival of 16 months in responders. In NSCLC, fibroblast activation protein (FAP)-targeted agents such as [^177^Lu]Lu-FAP-2286 demonstrated partial metabolic responses, including a 44.4% response rate and 78% disease control in a mixed cohort. Severe toxicities were infrequent.

**Discussion:**

TRT is a promising but experimental option for advanced lung cancer. Early efficacy signals exist for strong somatostatin receptor (SSTR)-targeted therapy in SCLC and FAP-targeted therapy in NSCLC, but evidence remains limited. Prospective trials with standardized protocols and dosimetry are needed to define TRT’s role in lung cancer treatment.

## Introduction

Lung cancer remains a major global health challenge and the leading cause of cancer-related mortality worldwide. Data from the GLOBOCAN 2020 indicate that it accounted for nearly 1.8 million deaths in 2020, corresponding to approximately 18% of all cancer deaths [1]. Lung cancer is broadly classified histologically into two main entities, non-small cell lung cancer (NSCLC) and small cell lung cancer (SCLC). NSCLC accounts for approximately 85% of lung cancer cases, and SCLC, which is a more aggressive neuroendocrine malignancy, comprises about 15% of cases [2].

SCLC is characterized by rapid proliferation, early metastatic spread, initial chemosensitivity, but frequent relapse and poor long-term survival (often less than 7% five-year survival for extensive-stage disease) [3]. NSCLC is a heterogeneous group of tumors that includes the major histologic subtypes of adenocarcinoma and squamous-cell carcinoma. According to the World Health Organization (WHO) classification criteria, adenocarcinoma accounts for roughly 40% of lung cancers, and squamous-cell carcinoma about 30%. Compared with SCLC, NSCLC typically grows more slowly and often presents at a less advanced stage [4].

Therapeutic options for both SCLC and NSCLC have seen significant advances in recent years. NSCLC treatment has been notably enhanced by a range of targeted therapies that inhibit specific driver mutations such as EGFR, ALK, ROS1, MET, and HER2, alongside immune checkpoint inhibitors [5]. Additionally, combined approaches involving chemoradiation and immunotherapy have become standard in many cases, improving patient outcomes [5].

However, despite these advancements, many patients either experience disease progression during or before receiving these therapies or lack identifiable mutations that would allow targeted treatment. Consequently, many patients still lack effective precision medicine options [6].

In nuclear medicine, the paradigm of “theranostics”, combining diagnosis (via imaging) and therapy (via targeted radionuclide delivery), has gained momentum [7, 8]. A targeted radioligand therapy (TRT) agent typically consists of a tumor-targeting ligand, a radionuclide, and a chelator/linker that binds them [9, 10]. With such an approach, one can identify target expression by imaging and then deliver cytotoxic radiation via the same or a similar vector.

Clinical success in other tumor types, most notably well-differentiated neuroendocrine tumors (NETs), including lung carcinoids treated with [^177^Lu]Lu-DOTATATE, and prostate cancer managed with [^177^Lu]Lu-PSMA-617, has firmly validated the therapeutic concept of TRT [9, 11, 12]. In well-differentiated NETs, peptide receptor radionuclide therapy (PRRT) is an established and highly effective modality, with efficacy closely linked to strong somatostatin receptor (SSTR) expression, as confirmed by [^68^Ga]Ga-DOTATATE positron emission tomography/computed tomography (PET/CT). Across clinical trials and real-world cohorts, PRRT consistently achieves high disease control rates, driven by both objective tumor responses and prolonged stabilization, while also improving progression-free survival (PFS) and providing durable symptom relief [13]. This raises the question: Could TRT also play a meaningful role in both SCLC and NSCLC?

In this systematic review, we evaluate the role of TRTs in both SCLC and NSCLC, summarize their mechanisms, evidence, and discuss clinical and translational challenges and opportunities.

## Materials and methods

This review was reported according to the Preferred Reporting Items for Systematic reviews and Meta-Analyses (PRISMA) guidelines [14], and was conducted in accordance with established methodological frameworks for systematic reviews and meta-analyses [15, 16]. The protocol for this systematic review has been registered in the International Prospective Register of Systematic Reviews (PROSPERO); registration number (CRD420251238530) [17]. The completed PRISMA 2020 checklist is provided in Table S1.

### Search strategy

Three electronic databases (PubMed, Embase and Scopus) were systematically searched from 2000 to November 2025 using the following terms: (“lung neoplasms” OR “lung cancer” OR “non small cell lung cancer” OR “nsclc” OR “small cell lung cancer” OR “sclc”) AND (“theranostics” OR “theranostic” OR “theragnostic” OR “radioligand therapy” OR “targeted radionuclide therapy” OR “prrt” OR “rlt” OR “molecular radiotherapy”) NOT (“carcinoid tumor” OR “carcinoid” OR “pulmonary neuroendocrine tumors” OR “pulmonary neuroendocrine”). The final search covered studies published up to November 2025. The search strategy was adapted for each database as appropriate.

The exclusion of “carcinoid” and “pulmonary neuroendocrine” was intentional to maintain a focused scope on high-grade lung cancers (SCLC and NSCLC), where theranostics remains investigational. The complete search strategy was adapted for each database using controlled vocabulary and keyword combinations, and the final search was conducted in November 2025 without restrictions on study design.

### Eligibility criteria

To ensure the inclusion of relevant studies, the following eligibility criteria were applied: only original research articles, clinical trials, case series, and case reports focusing on theranostics in SCLC and NSCLC were included. Studies involving patients diagnosed with any subtype of lung cancer treated with TRT were considered. We excluded reviews, editorials, conference abstracts, commentaries, animal studies, and incomplete studies. Only studies published in English were considered to ensure the accuracy and consistency of data extraction.

### Screening and data extraction

Two authors conducted the initial screening by evaluating the titles and abstracts of the records in Rayyan AI [18]. Following this, they independently performed a secondary screening by thoroughly reviewing the full text of the identified studies based on predetermined inclusion criteria.

Before screening, duplicate records were removed using both automated database tools. The screening process followed a two-stage approach (title/abstract screening followed by full-text review) performed independently by two reviewers.

Subsequently, data were independently extracted from the included studies using a predesigned Microsoft Excel spreadsheet. The data extraction form was pilot-tested on a subset of studies to ensure consistency between reviewers before full extraction was performed.

The extracted data included study identifiers (title, author, year, country/region, design) and patient characteristics such as cancer type, histological subtype, sample size, number receiving TRT, age, sex, Eastern Cooperative Oncology Group (ECOG) status, prior treatments, and disease stage. Diagnostic variables comprised the PET/CT radiotracer, relevant quantitative parameters (e.g., standardized uptake value (SUV) thresholds, lesion-to-liver or lesion-to-parotid ratios), and expression positivity criteria. Therapeutic data included the radioligand used, administered activity, number of cycles, intervals, cumulative dose, treatment setting, and therapy selection criteria. Clinical outcomes captured imaging response based on Response Evaluation Criteria in Solid Tumors Version 1.1 (RECIST 1.1) [19], PET Response Criteria in Solid Tumors (PERCIST) [20], or other reported criteria, along with PFS, overall survival (OS), time-to-progression, and symptom or quality-of-life changes. Safety outcomes included hematologic and non-hematologic toxicities and serious adverse events (AEs), while dosimetry data encompassed organ doses, absorbed dose estimates, and the dosimetry method used.

The reported adverse effects or toxicities according to the Common Terminology Criteria for Adverse Events (CTCAE) were also extracted. This criterion is a standardized system developed by the National Cancer Institute (NCI) to classify and grade the severity of AEs in patients undergoing cancer therapy. This system provides consistent terminology and a grading scale from 1 to 5, where grade 1 indicates mild events, and grade 5 represents fatal outcomes [21].

The extracted data was reviewed and validated by the reviewers to ensure accuracy and completeness. Disagreements during the data extraction process were resolved through discussion, and a third reviewer was consulted if necessary. This meticulous approach aimed to ensure the reliability and consistency of the data extracted for the systematic review.

### Risk of bias and quality assessment

Risk of bias in each clinical study included in the systematic review was assessed using the Risk Of Bias In Non-randomized Studies—of Interventions (ROBINS-I) tool [22]. ROBINS-I evaluates the potential bias across seven domains: (1) bias due to confounding, (2) bias in selection of participants, (3) bias in classification of interventions, (4) bias due to deviations from intended interventions, (5) bias due to missing data, (6) bias in measurement of outcomes and (7) bias in selection of the reported result. Each domain was assessed individually with judgments categorized as low, moderate, serious, critical risk of bias, or no information, depending on the study design. Risk-of-bias assessment was performed independently by two researchers, and any disagreements were resolved through discussion with a third researcher.

**Figure 1:**
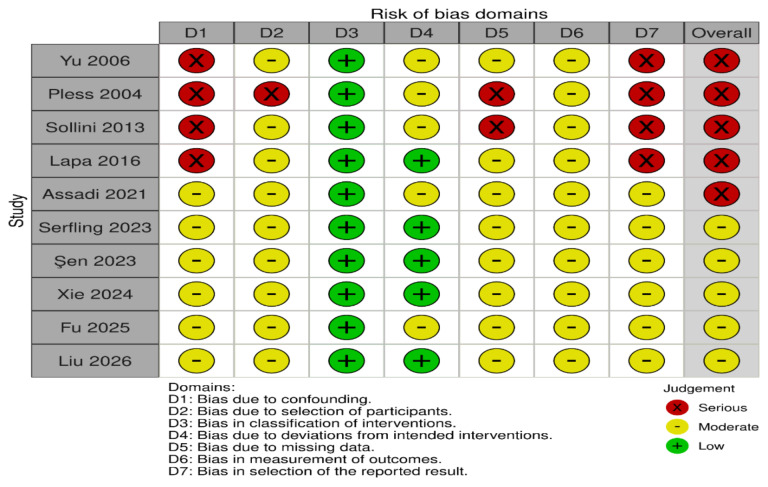
Risk Of Bias in Non-randomized Studies—of Interventions (ROBINS-I) assessment for included studies.

For each domain, signaling questions were used to guide the judgment, and the overall risk of bias for each study was determined based on the answers provided. The assessment was conducted using Excel, and Figure 1 was created using RobVis * with an additional reviewer brought in to resolve any potential disagreements between the primary reviewers.

## Results

### Study selection

**Figure 2:**
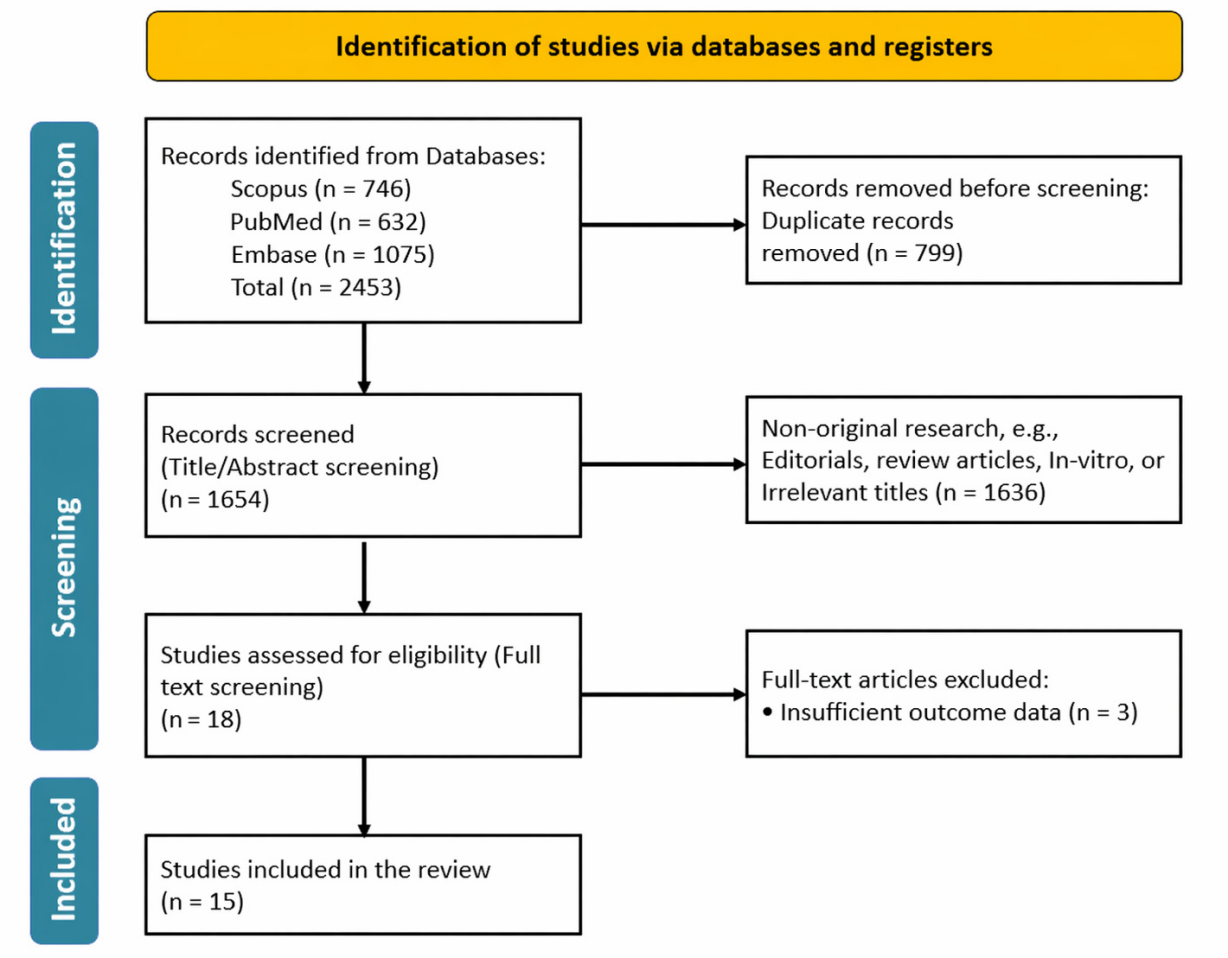
**Study selection process illustrated using the Preferred Reporting Items for Systematic Reviews and Meta-Analyses (PRISMA) 2020 flow diagram.** The diagram shows the identification, screening, eligibility, and inclusion steps of the literature search. Adapted from [14]. Accessed Nov 25, 2025. © 2024–2026 the PRISMA Executive. Distributed under a Creative Commons CC BY 4.0 license.

The initial search identified 2,453 published articles, from which 799 duplicates were removed, leaving 1,654 records for screening. Following the title and abstract evaluation, 1,636 studies were excluded as they did not meet the predefined inclusion criteria. During full-text assessment, an additional 3 articles were excluded due to insufficient data. Ultimately, 15 studies were included in the review, comprising 7 prospective clinical trials/prospective cohorts [23–29], 3 retrospective cohorts [30–32], 4 case reports [33–36], and 1 case series [37]. (Figure 2).

### Characteristics of the included studies

The studies were published between 2004 and 2025 and were conducted predominantly in Europe (Germany [30–32], Italy [26], Switzerland [25], and the Netherlands [37]) and Asia (China [23, 27–29, 34, 35], India [33], and Iran [24], Turkey [36]). Most were single-center experiences.

Across all 15 studies, a total of 358 lung cancer patients were reported across studies, of whom 105 received TRT. SCLC-focused cohorts accounted for 7 studies [25, 26, 30–32, 36, 37], with 250 SCLC patients overall and 67 SCLC patients treated with PRRT. NSCLC-specific cohorts were reported in 5 studies [28, 29, 33–35], together comprising 45 NSCLC patients, of whom 6 received TRT.

Three studies enrolled mixed or not-otherwise-specified lung cancer populations. Xie et al. [23] evaluated ^177^Lu-FAP-2286 efficacy in 9 patients (8 NSCLC and 1 SCLC) with advanced lung cancer. Yu et al. [27] reported on 43 patients (37 NSCLC and 6 SCLC) treated with [^131^I]Iodine-chTNT, and Assadi et al. [24] included 18 patients with various tumors, of whom 1 had non-reported histology lung cancer and received ^177^Lu-FAP-46.

Three primary theranostic targets were investigated across the included studies: SSTR predominantly and [^131^I]Iodine-chTNT in SCLC, fibroblast activation protein (FAP) in both SCLC and NSCLC, and prostate-specific membrane antigen (PSMA) in NSCLC. The majority of patients were heavily pre-treated with advanced (Stage IV) disease, indicating an application in the salvage or palliative setting. Heterogeneity in targets (SSTR, FAP, PSMA, TNT), radionuclides, dosing schedules, outcome definitions, and inclusion criteria precluded quantitative meta-analysis. The characteristics of all included studies are summarized in Table 1.

**Table 1:** Characteristics of included studies.

**First author (year)**	**Country**	**Study design**	**Cancer type**	**Sample size (treated)**	**Target**	**Diagnostic agent**	**Therapeutic agent**	**Treatment setting**
Pless, 2004 [25]	Switzerland	Pilot trial	SCLC	27 (6)	SSTR	[^111^In]In-octreoscan	[^90^Y]Y-DOTATOC	Salvage
van Essen, 2006 [37]	Netherlands	Case series	SCLC	3 (3)	SSTR	[^111^In]In-octreotide	[^177^Lu]Lu-DOTA-Tyr^3^-octreotate	Salvage
Yu, 2006 [27]	China	Phase IIa trial	SCLC and NSCLC	43 (22)	DNA/Histone	[^131^I]Iodine-chTNT	[^131^I]Iodine-chTNT	Palliative
Sollini, 2013 [26]	Italy	Prospective trial	SCLC	24 (11)	SSTR	[^68^Ga]Ga-DOTATOC/TATE	[^90^Y]Y/^177^Lu-DOTATOC/TATE	Salvage
Lapa, 2016 [31]	Germany	Retrospective cohort	SCLC	21 (4)	SSTR	[^68^Ga]Ga-DOTATATE	[^177^Lu]Lu-DOTATATE/TOC	Salvage
Assadi, 2021 [24]	Iran	Prospective study	Not specified	18 (1)	FAP	[^177^Lu]Lu-FAPI-46	[^177^Lu]Lu-FAPI-46	Palliative
Rao, 2023 [34]	China	Case report	NSCLC	1 (1)	FAP	[^68^Ga]Ga-FAP-2286	[^177^Lu]Lu-FAP-2286	Palliative
Şen, 2023 [32]	Germany	Retrospective cohort	SCLC	67 (14)	SSTR	[^68^Ga]Ga-DOTATOC	[^177^Lu]Lu-DOTATATE/TOC	Palliative
Serfling, 2023 [30]	Germany	Retrospective cohort	SCLC	100 (28)	SSTR	[^68^Ga]Ga-DOTATOC	PRRT (Not Specified)	Salvage
Yang, 2024 [35]	China	Case report	NSCLC	1 (1)	FAP	[^68^Ga]Ga-FAP-2286	[^177^Lu]Lu-FAP-2286	Palliative
Xie, 2024 [23]	China	Prospective cohort	SCLC and NSCLC	9 (9)	FAP	[^68^Ga]Ga-FAP-2286	[^177^Lu]Lu-FAP-2286	Palliative
Aggarwal, 2026 [33]	India	Case report	NSCLC	1 (1)	PSMA	[^68^Ga]Ga-PSMA-11	[^177^Lu]Lu-PSMA-617	Salvage
Liu, 2026 [28]	China	Phase I trial	NSCLC	14 (1)	FAP	[^68^Ga]Ga-FAPI	[^177^Lu]Lu-FAPI-XT	Salvage
Fu, 2025 [29]	China	Phase II trial	NSCLC	28 (2)	FAP	[^68^Ga]Ga-FAP-46	[^177^Lu]Lu-LNC1004	Salvage
Tuncel, 2025 [36]	Turkey	Case report	SCLC	1 (1)	SSTR	[^18^F]FDG and [^68^Ga]Ga-DOTATATE	[^225^Ac]Ac-DOTATATE	Salvage

FAP: fibroblast activation protein; NSCLC: non-small cell lung cancer; PSMA: prostate-specific membrane antigen; SCLC: small cell lung cancer; SSTR: strong somatostatin receptor.

### Risk of bias and quality assessment

Across the included studies, most early-phase and theranostic investigations demonstrated an overall moderate to serious risk of bias, which is expected for non-randomized, exploratory TRT trials. Many studies were prospective with clearly defined interventions, resulting in low risk for intervention classification and low-to-moderate risk for deviations from intended interventions. Key limitations were related to confounding and participant selection, reflecting the enrollment of heavily pre-treated, advanced-stage patients with high target expression. Missing data and outcome measurement were generally at moderate risk, since follow-up durations were short and blinding was not typically performed. Importantly, most studies lacked pre-registered statistical analysis plans, resulting in a moderate risk for selective reporting. Overall, the risk-of-bias profile is typical and acceptable for early-phase theranostic research, supporting cautious but meaningful interpretation of the therapeutic signal. The risk-of-bias rationale is presented in Table S2.

### Qualitative assessment

#### SCLC—somatostatin receptor therapy

Across the seven PRRT studies reporting therapeutic outcomes in SCLC, clinical activity was generally limited, with meaningful disease control observed only in small subsets of patients (Table 2). The largest cohort (*n* = 28) by Serfling et al. [30] did not provide detailed dosimetry, dosing, or efficacy metrics, limiting interpretability, but it showed that SSTR-PET changed management toward PRRT in 63.6% of cases with therapy change. Among studies with complete reporting, Lapa et al. [31] described the most favorable outcomes: Four patients treated with [^177^Lu]Lu-DOTATATE/TOC (7.6 ± 0.3 GBq per cycle, 1–6 cycles) achieved a 50% disease-control rate [1 partial response (PR), 1 stable disease (SD)], with notably prolonged median PFS (11.9 months) and OS (16 months) in responders, and no major toxicity. Şen et al. [32] (*n* = 14) similarly used [^177^Lu]Lu-DOTATATE/TOC (7.5 GBq, 1–6 cycles) but observed a lower disease-control rate of 38.5% and a much shorter median PFS of 3.6 months, again with no significant treatment-related toxicity. In contrast, studies using [^90^Y]Y-based PRRT showed uniformly poor outcomes. Sollini et al. [26] (*n* = 11) reported 0% disease control despite mixed [^90^Y]Y/[^177^Lu]Lu-DOTATOC/TATE regimens (2.6 GBq and 6.0 GBq, respectively), with grade 2–3 hematologic toxicity. Similarly, Pless et al. [25] (*n* = 6) reported progressive disease in all patients treated with [^90^Y]Y-DOTATOC (2.22 GBq/m^2^; median two cycles), with very short median PFS (1.3 months) and OS (3.6 months), and only mild anemia. van Essen et al. [37] (*n* = 3) also observed no disease control with [^177^Lu]Lu-DOTA-Tyr3-octreotate (7.4 GBq, 1–2 cycles), with transient liver enzyme elevation as the main toxicity. Overall, [^177^Lu]Lu-labelled agents demonstrated a more favorable safety profile and occasional disease stabilization, whereas [^90^Y]Y-based therapy was associated with higher hematologic toxicity and no objective responses across studies. Notably, a recent case report by Tuncel et al. [36] described the first documented use of α-PRRT in chemotherapy-refractory SCLC: A 65-year-old man with high SSTR expression received a single cycle of [^225^Ac]Ac-DOTATATE (9 MBq), achieving a marked partial metabolic response with only faint residual uptake on post-therapy [^18^F]FDG and [^68^Ga]Ga-DOTATATE PET/CT.

**Table 2:** Characteristics and outcomes of PRRT studies in SCLC.

**Study (first author, year)**	**Sample size**	**Therapeutic agent(s)**	**Activity per cycle (GBq)**	**Number of cycles**	**Response criteria**	**Response rate (PR+SD)**	**Median PFS (months)**	**Median OS (months)**	**Major toxicity**
Serfling, 2023 [30]	28	Not specified PRRT	Not reported	Not reported	Not reported	Not reported	Not reported	Not reported	Not reported
Lapa, 2016 [31]	4	[^177^Lu]Lu-DOTATATE/TOC	7.6 ± 0.3	1–6	Not reported	50% (1 PR,1 SD)	11.9 (responders)	16 (responders)	None
Sollini, 2013 [26]	11	[^90^Y]Y/[^177^Lu]Lu-DOTATOC/TATE	2.6 (Y), 6.0 (Lu)	1–3	RECIST 1.1	0% (all PD)	NA	NA	G2-3 hematologic
Şen, 2023 [32]	14	[^177^Lu]Lu-DOTATATE/TOC	7.5	1–6	RECIST 1.1	38.5% (PR+SD)	3.6	Not reported	None
Pless, 2004 [25]	6	[^90^Y]Y-DOTATOC	2.22 (60 mCi/m^2^)	Median 2	WHO	0% (all PD)	1.3	3.6	G1-2 anemia
van Essen, 2006 [37]	3	[^177^Lu]Lu-DOTA-Tyr3-Octreotate	7.4	1–2	SWOG	0% (all PD)	NA	NA	Liver enzyme elevation
Tuncel, 2025 [36]	1	[^225^Ac]Ac-DOTATATE	0.009 (9 MBq)	1	Not reported	100% (1 PR)	Not reported	Not reported	Not reported

NA: not applicable; OS: overall survival; PD: progressive disease; PFS: progression-free survival; PR: partial response; PRRT: peptide receptor radionuclide therapy; RECIST 1.1: Response Evaluation Criteria in Solid Tumors Version 1.1; SCLC: small cell lung cancer; SD: stable disease; SWOG: Southwest Oncology Group; WHO: World Health Organization.

#### NSCLC—FAPI and PSMA therapy

Across the five small studies and case reports evaluating TRT in NSCLC, therapeutic activity and clinical responses varied substantially depending on the agent used and underlying tumour biology (Table 3). Three single-patient case reports using [^177^Lu]Lu-FAP agents [28, 34, 35] demonstrated heterogeneous outcomes: While [^177^Lu]Lu-FAP-2286 produced partial metabolic responses after a single 7.0–7.4 GBq cycle in two patients, [^177^Lu]Lu-FAPI-XT (11.1 GBq) resulted in early progression within 0.75 months, suggesting that not all FAP-targeting constructs exhibit comparable treatment performance. Two lung-cancer patients treated with [^177^Lu]Lu-LNC1004 were part of a broader prospective cohort of 28 patients representing 11 malignancies, among whom disease control was achieved in 46% (4 PR, 9 SD); however, the study did not provide lung-cancer-specific outcomes, limiting interpretation for this subgroup. In this mixed-cancer cohort, grade 3/4 hematologic toxicity occurred in 21% (primarily thrombocytopenia, leukopenia, and neutropenia), with no high-grade hepatic or renal toxicity reported [29]. A single NSCLC case treated with a low activity dose of [^177^Lu]Lu-PSMA-617 [33] did not provide response or survival outcomes, however, detailed dosimetric analysis demonstrated a very low tumor-absorbed dose of 0.74 Gy/GBq, with higher uptake in normal organs including the salivary glands (6.24 Gy/GBq), liver (2.28 Gy/GBq), spleen (3.42 Gy/GBq), and kidneys (1.71 Gy/GBq), reflecting rapid radiotracer washout from the tumor and insufficient energy deposition for therapeutic effect.

**Table 3:** Characteristics and outcomes of TRT studies in NSCLC.

**Study (first author, year)**	**Sample size**	**Therapeutic agent**	**Activity per cycle (GBq)**	**Number of cycles**	**Response criteria**	**Response**	**Median PFS (months)**	**Median OS (months)**	**Major toxicity**
Rao, 2023 [34]	1	[^177^Lu]Lu-FAP-2286	7	1	PERCIST	PR	Not reported	Not reported	None
Yang, 2024 [35]	1	[^177^Lu]Lu-FAP-2286	7.4	1	PERCIST	PR	Not reported	Not reported	None
Liu, 2026 [28]	1	[^177^Lu]Lu-FAPI-XT	11.1	1	RECIST 1.1	PD	0.75	Not reported	None
Fu, 2025 [29]	2	[^177^Lu]Lu-LNC1004	3.33	Not specified	RECIST 1.1	Not reported	Not reported	Not reported	Not reported
Aggarwal, 2026 [33]	1	[^177^Lu]Lu-PSMA-617	0.466 (single dose)	1	Not reported	Not reported	Not reported	Not reported	Not reported

NSCLC: non-small cell lung cancer; OS: overall survival; PD: progressive disease; PERCIST: positron emission tomography Response Criteria in Solid Tumors; PFS: progression-free survival; PR: partial response; RECIST 1.1: Response Evaluation Criteria in Solid Tumors Version 1.1; TRT: targeted radioligand therapy.

#### Mixed SCLC and NSCLC populations

Two studies evaluated TRT approaches in a mixed population of SCLC and NSCLC, demonstrating heterogeneous but clinically meaningful outcomes (Table 4). In a prospective cohort of nine patients treated with [^177^Lu]Lu-FAP-2286, Xie et al. [23] reported a mean of 3.1 treatment cycles (range, 2–6), achieving a partial metabolic response rate of 44.4% and an overall disease-control rate of 78%, alongside improvements in quality of life and no grade III/IV toxicities, supporting the agent’s favorable tolerability profile. In contrast, the larger radioimmunotherapy cohort by Yu et al. [27], which included 43 patients receiving two cycles of [^131^I]Iodine-chTNT, demonstrated modest systemic responses (9.1% partial/complete response). Although the original trial also explored intratumoral administration of [^131^I]Iodine-chTNT, only the systemic approach was included in this review to maintain consistency with the intravenous, whole-body radioligand therapies evaluated. Hematologic toxicity in Yu et al. [27] was generally mild to moderate based on WHO criteria, and survival outcomes were not reported, whereas Xie et al. [23] documented a median PFS of 6 months and a median OS of 10 months.

**Table 4:** Characteristics and outcomes of TRT studies in SCLC and NSCLC.

**Study (first author, year)**	**Sample size**	**Therapeutic agent**	**Number of cycles**	**Response criteria**	**Response rate (PR+SD)**	**Median PFS (months)**	**Median OS (months)**	**Major toxicity**
Xie, 2024, [23]	9	[^177^Lu]Lu-FAP-2286	Mean 3.1 (2–6)	RECIST 1.1	44.4%	6	10	Mild (no grade III/IV)
Yu, 2006, [27]	43	[^131^I]Iodine-chTNT	2	WHO	9.1%	Not reported	Not reported	Mild to moderate hematologic toxicity

NSCLC: non-small cell lung cancer; OS: overall survival; PD: progressive disease; PFS: progression-free survival; PR: partial response; SD: stable disease; RECIST 1.1: Response Evaluation Criteria in Solid Tumors Version 1.1; SCLC: small cell lung cancer; TRT: targeted radioligand therapy; WHO: World Health Organization.

#### Not specified-histology populations

Assadi et al. [24] reported a preliminary prospective study evaluating [^177^Lu]Lu-FAPI-46 in a cohort of patients with relapsed or refractory solid tumors, which included one lung cancer patient, although the authors did not specify whether this case was SCLC or NSCLC. The patient received four cycles of 3.7 GBq administered at 4–6-week intervals, following confirmation of adequate tumor uptake on [^177^Lu]Lu-FAPI-46 scintigraphy. According to RECIST 1.1, this patient achieved SD with a follow-up duration of 4.5 months, reflecting disease stabilization in a heavily pretreated setting. No hematologic, non-hematologic, or serious AEs were reported, consistent with the overall favorable safety profile observed in the wider study population.

## Discussion

To the best of our knowledge, this systematic review is the first to comprehensively synthesize the evolving evidence for TRT across both major histological subtypes of lung cancer, SCLC and NSCLC. Our analysis of fifteen studies [23–37], encompassing 105 patients who received TRT, reveals a rapidly developing field characterized by promising yet preliminary findings. The therapeutic strategies are distinctly bifurcated: PRRT has been the primary focus in SCLC, while emerging research in NSCLC explores FAP and, to a lesser extent, PSMA as novel targets.

Regarding clinical efficacy, PRRT outcomes in SCLC were heterogeneous but demonstrated that disease control may be achievable in a subset of patients. Disease control rates for SSTR-targeted therapy ranged from 0% to 50% across studies [25, 26, 31, 32, 37]. Notably, the most favorable outcomes were reported by Lapa et al. [31], where two of four treated patients achieved prolonged disease stabilization (median PFS 11.9 months) with [^177^Lu]Lu-DOTATATE/TOC. In contrast, studies utilizing [^90^Y]Y-based agents uniformly reported 0% disease control and were associated with higher rates of hematologic toxicity [25, 26]. Emerging evidence from alpha-emitter PRRT further supports the theranostic rationale in SCLC; a recent case by Tuncel et al. [36] demonstrated a marked partial metabolic response following a single cycle of [^225^Ac]Ac-DOTATATE in a chemotherapy-refractory patient. This suggests that the choice of radionuclide is a critical determinant of both efficacy and safety, with [^177^Lu]Lu potentially offering a more favorable therapeutic index in this setting, while alpha-emitter strategies may represent a promising next-generation alternative.

In NSCLC, the emerging data on FAP-targeted therapy are particularly biologically interesting with early signals of activity, albeit based on a very small number of patients. Two case reports using [^177^Lu]Lu-FAP-2286 demonstrated partial metabolic responses after a single cycle, indicating potent biological activity [34, 35]. This is supported by the larger mixed-cohort study by Xie et al. [23], where [^177^Lu]Lu-FAP-2286 achieved a 44.4% PR rate and a 78% disease control rate in a population of advanced SCLC and NSCLC patients. Conversely, the single case of NSCLC treated with [^177^Lu]Lu-PSMA-617 revealed a fundamental pharmacokinetic challenge, with a low tumor-absorbed dose (0.74 Gy/GBq) due to rapid washout, underscoring that target expression on imaging does not always guarantee therapeutic efficacy [33].

Importantly, TRT demonstrated a generally favorable safety profile in lung cancer patients, particularly for [^177^Lu]Lu-based agents. Across the included studies, only 3 patients had severe (grade 3/4) toxicities. The most common AEs were mild to moderate hematologic toxicities, which were generally manageable. This favorable tolerability is a significant advantage for heavily pre-treated patients in the salvage or palliative settings where these therapies are currently being applied.

None of the included studies directly compared TRT with standard systemic treatments, underscoring a significant gap in the current research landscape. Moreover, the vast majority of patients in these studies had undergone extensive pretreatment with chemotherapy, immunotherapy, and/or radiotherapy before receiving TRT. Therefore, investigating the efficacy of these novel theranostic approaches in earlier lines of therapy or in selected patient populations with high target expression could offer valuable insights into their potential role in the treatment paradigm.

The predictive value of baseline SSTR-, FAP-, or PSMA-directed PET for subsequent therapeutic efficacy remains an area of active investigation. While SSTR-PET was shown to change management towards PRRT in a majority of SCLC patients in one study [30], and FAP-PET reliably identifies FAP-expressing tumors, their capacity to forecast therapeutic outcomes is multifaceted and not yet fully elucidated. A key challenge, particularly for FAP- and PSMA-targeting agents in NSCLC, is the rapid washout of these compounds from tumor tissues, which can diminish the delivered radiation dose [28, 33]. It is anticipated that baseline PET uptake alone is insufficient to predict TRT efficacy. Tumor retention kinetics, internalization rates, and absorbed doses appear far more critical than qualitative visual assessment or static SUV measurements. A notable finding emerging from this review is the potential value of combining TRT with complementary treatment modalities. In the radioimmunotherapy study by Yu et al. [27], the intratumoral arm of [^131^I]Iodine-chTNT, although not included in the present analysis, which focused exclusively on the systemic approach, demonstrated a markedly higher intratumoral response rate (56%) compared with the modest systemic response rate (9.1%). This differential pattern underscores a distinct mechanism of action driven by direct tumoral retention of the antibody construct, suggesting that locoregional delivery may offer synergistic potential when integrated with systemic or multimodal regimens.

Regarding radionuclide selection, our findings in SCLC suggest that [^177^Lu]Lu may be preferable to [^90^Y]Y due to its more favorable safety profile and observed instances of efficacy. For novel targets such as FAP-, addressing the challenge of rapid ligand washout is critical for effective radionuclide therapy. Radionuclides with shorter or more compatible half-lives, such as terbium-161 [^161^Tb]Tb [38], show promise because of their emission characteristics, including beta particles and densely ionizing Auger electrons that can increase tumor cell kill at micro dosimetric levels [38].

Given the extremely limited and heterogeneous evidence base, TRT in lung cancer should currently be regarded as an experimental therapeutic approach. Its use ought to be restricted to prospective clinical trials or, in exceptional circumstances, highly selected salvage cases managed in experienced centers with access to individualized dosimetry, multidisciplinary evaluation, and close post-therapy monitoring. At present, there is no evidence to support the routine or first-line use of TRT in either SCLC or NSCLC outside a research framework.

It is important to indicate that this review has several limitations. First, the number of patients treated with TRT, particularly for NSCLC, is very small, and the evidence is dominated by retrospective cohorts and case reports, which are susceptible to publication bias. Significant variability exists across the included studies, with marked differences in patient populations, therapeutic protocols, radionuclides, and outcome reporting, which complicates the ability to conduct a pooled quantitative analysis. The almost universal application of TRT in a late-line, heavily pretreated setting limits generalizability to earlier disease stages. Consequently, it is imperative to conduct additional prospective studies with larger sample sizes, standardized protocols, and extended follow-up durations to enhance our understanding of the intervention’s efficacy, optimal implementation, and long-term safety in both SCLC and NSCLC.

### Future perspectives

Although several innovative molecular targets are being explored in lung cancer, none of the currently emerging tracers have yet been translated into established radioligand therapies for either SCLC or NSCLC. Among the most advanced are hypoxia-targeted agents, such as ^18^F-HX4, which allow non-invasive characterization of intertumoral hypoxia, a key driver of radioresistance and progression in lung cancer, yet remain strictly investigational and limited to diagnostic imaging rather than therapeutic use [39]. Integrin αvβ6-targeted tracers, including ^68^Ga-Trivehexin, have shown highly selective epithelial tumor binding in NSCLC and offer potential future theranostic utility, but currently exist only as imaging probes with no paired therapeutic isotopes in clinical development [40]. Another target under investigation is the gastrin-releasing peptide receptor (GRPR), where compounds such as NeoB have demonstrated tumor binding in early studies; however, therapeutic radiolabeled analogues like ^177^Lu-NeoB remain experimental and have not yet been validated in lung cancer populations [41].

### Conclusion

TRT for lung cancer remains investigational, with current evidence limited to small, heterogeneous early-phase studies. In SCLC, beta-emitter [^177^Lu]Lu and alpha-[^225^Ac]Ac-based PRRT demonstrated considerable disease stabilization with acceptable safety, whereas beta-emitter [^90^Y]Y-based therapy showed minimal efficacy and higher toxicity. In NSCLC, early experiences with FAP-targeted agents suggest potential biological activity, while PSMA-targeted therapy appears therapeutically insufficient despite imaging uptake. Overall, the small sample sizes, non-comparative designs, and high risk of bias preclude firm conclusions regarding efficacy. Well-designed prospective trials incorporating standardized imaging, dosimetry, and target selection are essential before TRT can be integrated into the treatment landscape for SCLC or NSCLC.

## Abbreviations

AEs: adverse events
FAP: fibroblast activation protein
NETs: neuroendocrine tumors
NSCLC: non-small cell lung cancer
OS: overall survival
PET: positron emission tomography
PET/CT: positron emission tomography/computed tomography
PFS: progression-free survival
PR: partial response
PRISMA: Preferred Reporting Items for Systematic reviews and Meta-Analyses
PRRT: peptide receptor radionuclide therapy
PSMA: prostate-specific membrane antigen
RECIST 1.1: Response Evaluation Criteria in Solid Tumors Version 1.1
SCLC: small cell lung cancer
SD: stable disease
SSTR: strong somatostatin receptor
SUV: standardized uptake value
TRT: targeted radioligand therapy

## Supplementary materials

The supplementary tables for this article are available at: https://www.explorationpub.com/uploads/Article/file/1002368_sup_1.pdf.

## Declarations

### Availability of data and materials

All data generated or analyzed during this study are included in the manuscript.

### Copyright

© The Author(s) 2026.

## References

[1] Bray F, Laversanne M, Sung H, Ferlay J, Siegel RL, Soerjomataram I (2024). Global cancer statistics 2022: GLOBOCAN estimates of incidence and mortality worldwide for 36 cancers in 185 countries. CA Cancer J Clin.

[2] Inamura K (2017). Lung Cancer: Understanding Its Molecular Pathology and the 2015 WHO Classification. Front Oncol.

[3] Blackhall F, Girard N, Livartowski A, McDonald L, Roset M, Lara N (2023). Treatment patterns and outcomes among patients with small-cell lung cancer (SCLC) in Europe: a retrospective cohort study. BMJ Open.

[4] Liang B, Tong C, Nong J, Zhang Y (2024). Histological Subtype Classification of Non-Small Cell Lung Cancer with Radiomics and 3D Convolutional Neural Networks. J Imaging Inform Med.

[5] Jeon H, Wang S, Song J, Gill H, Cheng H (2025). Update 2025: Management of NonSmall-Cell Lung Cancer. Lung.

[6] Niho S (2025). Treatment of small cell lung cancer; advances and future prospects. Respir Investig.

[7] Al-Ibraheem A, Zimmermann R, Abdlkadir AS, Herrmann K (2024). Radiotheranostics Global Market and Future Developments. Semin Nucl Med.

[8] Al-Ibraheem A, Brink A, Lee ST, De Los Reyes A, Paez D, Craviolatti PS (2025). Implementation of Radiotheranostics: Challenges, Barriers, and IAEA-Driven Strategies for Sustainable Access. Semin Nucl Med.

[9] Watabe T, Hirata K, Iima M, Yanagawa M, Saida T, Sakata A (2025). Recent advances in theranostics and oncology PET: emerging radionuclides and targets. Ann Nucl Med.

[10] Al-Ibraheem A, Scott AM, Abdlkadir AS, Vrachimis A, Lamoureux F, Trujillo PB (2025). Consensus Nomenclature for Radionuclide Therapy: Initial Recommendations from Nuclear Medicine Global Initiative. J Nucl Med.

[11] Abdlkadir AS, Al-Adhami D, Al-Rasheed U, Jreige M, Mahafza W, Al-Khawaldeh K (2025). PET imaging and radionuclide therapy in neuroendocrine prostate cancer: a systematic review. Q J Nucl Med Mol Imaging.

[12] Al-Ibraheem A, Abdlkadir AS, Sweedat DA, Maus S, Al-Rasheed U, Salah S (2024). From Despair to Hope: First Arabic Experience of ^177^Lu-PSMA and ^161^Tb-PSMA Therapy for Metastatic Castration-Resistant Prostate Cancer. Cancers (Basel).

[13] Naraev BG, Ramirez RA, Kendi AT, Halfdanarson TR (2019). Peptide Receptor Radionuclide Therapy for Patients With Advanced Lung Carcinoids. Clin Lung Cancer.

[14] Page MJ, McKenzie JE, Bossuyt PM, Boutron I, Hoffmann TC, Mulrow CD (2021). The PRISMA 2020 statement: an updated guideline for reporting systematic reviews. BMJ.

[15] Calderon Martinez E, Ghattas Hasbun PE, Salolin Vargas VP, García-González OY, Fermin Madera MD, Rueda Capistrán DE (2025). A comprehensive guide to conduct a systematic review and meta-analysis in medical research. Medicine (Baltimore).

[16] Selçuk AA (2019). A Guide for Systematic Reviews: PRISMA. Turk Arch Otorhinolaryngol.

[17] Saad Ruzzeh, Serin Moghrabi. The Evolving Role of Targeted Radioligand Therapy in Small Cell and Non-Small Cell Lung Cancer: A Systematic Review. PROSPERO 2025 CRD420251238530 [Internet]. https://www.crd.york.ac.uk/PROSPERO/view/CRD420251238530.

[18] Ouzzani M, Hammady H, Fedorowicz Z, Elmagarmid A (2016). Rayyan-a web and mobile app for systematic reviews. Syst Rev.

[19] Schwartz LH, Litière S, de Vries E, Ford R, Gwyther S, Mandrekar S (2016). RECIST 1.1-Update and clarification: From the RECIST committee. Eur J Cancer.

[20] O JH, Lodge MA, Wahl RL (2016). Practical PERCIST: A Simplified Guide to PET Response Criteria in Solid Tumors 1.0. Radiology.

[21] Freites-Martinez A, Santana N, Arias-Santiago S, Viera A (2021). Using the Common Terminology Criteria for Adverse Events (CTCAE - Version 5.0) to Evaluate the Severity of Adverse Events of Anticancer Therapies. Actas Dermosifiliogr (Engl Ed).

[22] Sterne JA, Hernán MA, Reeves BC, Savović J, Berkman ND, Viswanathan M (2016). ROBINS-I: a tool for assessing risk of bias in non-randomised studies of interventions. BMJ.

[23] Xie Y, Ma J, Tang W, Zhang Y, Zhang C, Chen Y (2024). Efficacy and Safety Evaluation of 177Lu-FAP-2286 in the Treatment of Advanced Lung Cancer. Clin Nucl Med.

[24] Assadi M, Rekabpour SJ, Jafari E, Divband G, Nikkholgh B, Amini H (2021). Feasibility and Therapeutic Potential of 177Lu-Fibroblast Activation Protein Inhibitor-46 for Patients With Relapsed or Refractory Cancers: A Preliminary Study. Clin Nucl Med.

[25] Pless M, Waldherr C, Maecke H, Buitrago C, Herrmann R, Mueller-Brand J (2004). Targeted radiotherapy for small cell lung cancer using 90Yttrium-DOTATOC, an Yttrium-labelled somatostatin analogue: a pilot trial. Lung Cancer.

[26] Sollini M, Farioli D, Froio A, Chella A, Asti M, Boni R (2013). Brief report on the use of radiolabeled somatostatin analogs for the diagnosis and treatment of metastatic small-cell lung cancer patients. J Thorac Oncol.

[27] Yu L, Ju DW, Chen W, Li T, Xu Z, Jiang C (2006). 131I-chTNT radioimmunotherapy of 43 patients with advanced lung cancer. Cancer Biother Radiopharm.

[28] Liu H, Guo R, Zhang X, Ji H, Sun S, Sun S (2026). Safety and efficacy of ^177^Lu-FAPI-XT radioligand therapy in patients with advanced sarcoma and other cancer entities: first-in-human, dose-escalation study. Eur J Nucl Med Mol Imaging.

[29] Fu H, Huang J, Zhao L, Chen Y, Xu W, Cai J (2025). 177Lu-LNC1004 Radioligand Therapy in Patients with End-stage Metastatic Cancers: A Single-Center, Single-Arm, Phase II Study. Clin Cancer Res.

[30] Serfling SE, Hartrampf PE, Zhi Y, Higuchi T, Kosmala A, Serfling J (2023). Somatostatin Receptor-Directed PET/CT for Therapeutic Decision-Making and Disease Control in Patients Affected With Small Cell Lung Cancer. Clin Nucl Med.

[31] Lapa C, Hänscheid H, Wild V, Pelzer T, Schirbel A, Werner RA (2016). Somatostatin receptor expression in small cell lung cancer as a prognostic marker and a target for peptide receptor radionuclide therapy. Oncotarget.

[32] Şen F, Sheikh GT, von Hinten J, Schindele A, Kircher M, Dierks A (2023). In-Vivo Somatostatin-Receptor Expression in Small Cell Lung Cancer as a Prognostic Image Biomarker and Therapeutic Target. Cancers (Basel).

[33] Aggarwal P, Anwariya A, Kaur K, Satapathy S, Sood A, Singh N (2026). PSMA-based Theranostics in Advanced Non-small Cell Lung Cancer: A Hit or a Miss?. Clin Nucl Med.

[34] Rao Z, Zhang Y, Liu L, Wang M, Zhang C (2023). [^177^Lu]Lu-FAP-2286 therapy in a case of right lung squamous cell carcinoma with systemic metastases. Eur J Nucl Med Mol Imaging.

[35] Yang H, Liu H, Zhang Y, Zhang Y, Chen Y (2024). Metastatic Lung Adenocarcinoma Received Combined 177 Lu-FAP-2286 Radiation Therapy and Targeted Therapy. Clin Nucl Med.

[36] Tuncel M, Türkan C, Eryılmaz Y, Pala A, Kılıçkap S (2025). Significant response to [^225^Ac]Ac-DOTATATE therapy in a patient with small cell lung cancer. Eur J Nucl Med Mol Imaging.

[37] van Essen M, Krenning EP, Kooij PP, Bakker WH, Feelders RA, de Herder WW (2006). Effects of therapy with [177Lu-DOTA0, Tyr3]octreotate in patients with paraganglioma, meningioma, small cell lung carcinoma, and melanoma. J Nucl Med.

[38] Abdlkadir AS, Rosar F, Jalilian A, Moghrabi S, Al-Balooshi B, Rabei O (2025). Harnessing Terbium Radioisotopes for Clinical Advancements: A Systematic Review. Nucl Med Mol Imaging.

[39] Verwer EE, Zegers CML, van Elmpt W, Wierts R, Windhorst AD, Mottaghy FM (2016). Pharmacokinetic modeling of a novel hypoxia PET tracer [^18^F]HX4 in patients with non-small cell lung cancer. EJNMMI Phys.

[40] Urso L, Napolitano R, Speltri G, Tuncel M, Badrane I, Uccelli L (2025). ^68^Ga-Trivehexin: Current Status of αvβ6-Integrin Imaging and Perspectives. Cancers (Basel).

[41] Ruigrok EAM, Verhoeven M, Konijnenberg MW, de Blois E, de Ridder CMA, Stuurman DC (2022). Safety of [^177^Lu]Lu-NeoB treatment: a preclinical study characterizing absorbed dose and acute, early, and late organ toxicity. Eur J Nucl Med Mol Imaging.

