# Supplementary material for: The evolving role of targeted radioligand therapy in small cell and non-small cell lung cancer: a systematic review
Source: Explor Target Antitumor Ther. 2026 Apr 27;7:1002368. doi: 10.37349/etat.2026.1002368 (PMC13129189; doi:10.37349/etat.2026.1002368)
Supplement: Supplementary file 1 [file 1002368_sup_1.pdf]

Table S1. PRISMA checklist.

Section and Topic	Item #	Checklist item	Location where item is reported
**TITLE**			
Title	1	Identify the report as a systematic review.	Identified as a systematic review (Title page; Abstract).
**ABSTRACT**			
Abstract	2	See the PRISMA 2020 for Abstracts checklist.	Structured abstract with Background, Methods, Results, Conclusions (Abstract, p. 1).
**INTRODUCTION**			
Rationale	3	Describe the rationale for the review in the context of existing knowledge.	Introduction, first three paragraphs (pp. 2–3).
Objectives	4	Provide an explicit statement of the objective(s) or question(s) the review addresses.	Introduction, last paragraph stating aims (p. 3).
**METHODS**			
Eligibility criteria	5	Specify the inclusion and exclusion criteria for the review and how studies were grouped for the syntheses.	Methods §2.2 Eligibility Criteria (p. 3).
Information sources	6	Specify all databases, registers, websites, organisations, reference lists and other sources searched or consulted to identify studies. Specify the date when each source was last searched or consulted.	Methods §2.1 Search Strategy—databases (PubMed, Embase, Scopus) (p. 3).
Search strategy	7	Present the full search strategies for all databases, registers and websites, including any filters and limits used.	Methods §2.1 Search Strategy—full boolean terms (p. 3).
Selection process	8	Specify the methods used to decide whether a study met the inclusion criteria of the review, including how many reviewers screened each record and each report retrieved, whether they worked independently, and if applicable, details of automation tools used in the process.	Methods §2.3 Screening and Data Extraction—title/abstract and full-text selection with Rayyan (p. 4).

Data collection process	9	Specify the methods used to collect data from reports, including how many reviewers collected data from each report, whether they worked independently, any processes for obtaining or confirming data from study investigators, and if applicable, details of automation tools used in the process.	Methods §2.3 Screening and Data Extraction—independent, duplicate extraction (p. 4).
Data items	10a	List and define all outcomes for which data were sought. Specify whether all results that were compatible with each outcome domain in each study were sought (e.g. for all measures, time points, analyses), and if not, the methods used to decide which results to collect.	Methods §2.3—variables extracted (p. 4).
	10b	List and define all other variables for which data were sought (e.g. participant and intervention characteristics, funding sources). Describe any assumptions made about any missing or unclear information.	Methods §2.3—variables extracted (p. 4).
Study risk of bias assessment	11	Specify the methods used to assess risk of bias in the included studies, including details of the tool(s) used, how many reviewers assessed each study and whether they worked independently, and if applicable, details of automation tools used in the process.	Methods §2.4 Risk of Bias—ROBINS-1 domains and approach (p. 4,5).
Effect measures	12	Specify for each outcome the effect measure(s) (e.g. risk ratio, mean difference) used in the synthesis or presentation of results.	Not applicable (no meta-analysis).
Synthesis methods	13a	Describe the processes used to decide which studies were eligible for each synthesis (e.g. tabulating the study intervention characteristics and comparing against the planned groups for each synthesis (item #5)).	Not applicable (no meta-analysis).
	13b	Describe any methods required to prepare the data for presentation or synthesis, such as handling of missing summary statistics, or data conversions.	Not applicable (no meta-analysis).
	13c	Describe any methods used to tabulate or visually display results of individual studies and syntheses.	Narrative presentation; study characteristics in Table 1; PRISMA flow diagram in Fig. 1 (Results §3.1).
	13d	Describe any methods used to synthesize results and provide a rationale for the choice(s). If meta-analysis was performed, describe the model(s), method(s) to identify the presence and extent of statistical heterogeneity, and software package(s) used.	Narrative synthesis described in Discussion §4 (pp. 12-14); no pooling performed.

	13e	Describe any methods used to explore possible causes of heterogeneity among study results (e.g. subgroup analysis, meta-regression).	Not applicable (no meta-analysis).
	13f	Describe any sensitivity analyses conducted to assess robustness of the synthesized results.	Not applicable (no meta-analysis).
Reporting bias assessment	14	Describe any methods used to assess risk of bias due to missing results in a synthesis (arising from reporting biases).	Not applicable (no meta-analysis).
Certainty assessment	15	Describe any methods used to assess certainty (or confidence) in the body of evidence for an outcome.	Not formally assessed; limitations and strength of evidence discussed in Discussion §4 (pp. 8-11).
**RESULTS**			
Study selection	16a	Describe the results of the search and selection process, from the number of records identified in the search to the number of studies included in the review, ideally using a flow diagram.	Results §3.1; PRISMA flow diagram (Figure 1) (p. 5).
	16b	Cite studies that might appear to meet the inclusion criteria, but which were excluded, and explain why they were excluded.	Results §3.1; PRISMA flow diagram (Figure 1) (p. 5).
Study characteristics	17	Cite each included study and present its characteristics.	Results §3.2; Table 1 (pp. 6-7).
Risk of bias in studies	18	Present assessments of risk of bias for each included study.	Results §3.3; Figure 2 (p. 8).
Results of individual studies	19	For all outcomes, present, for each study: (a) summary statistics for each group (where appropriate) and (b) an effect estimate and its precision (e.g. confidence/credible interval), ideally using structured tables or plots.	Results §3.4 (pp. 8-11) — individual study findings are described in detail for each included publication.
Results of syntheses	20a	For each synthesis, briefly summarise the characteristics and risk of bias among contributing studies.	Results §3.3 (p. 8) — ROBINS-1 evaluation summarized.
	20b	Present results of all statistical syntheses conducted. If meta-analysis was done, present for each the summary estimate and its precision (e.g. confidence/credible interval) and measures of statistical heterogeneity. If comparing groups, describe the direction of the effect.	Not applicable (qualitative synthesis only; no meta-analysis performed).

	20c	Present results of all investigations of possible causes of heterogeneity among study results.	Not applicable.
	20d	Present results of all sensitivity analyses conducted to assess the robustness of the synthesized results.	Not applicable.
Reporting biases	21	Present assessments of risk of bias due to missing results (arising from reporting biases) for each synthesis assessed.	Not applicable (no quantitative synthesis).
Certainty of evidence	22	Present assessments of certainty (or confidence) in the body of evidence for each outcome assessed.	Not assessed formally; discussed under Limitations (Discussion §4, p. 8-11
**DISCUSSION**			
Discussion	23a	Provide a general interpretation of the results in the context of other evidence.	Discussion §4 (pp. 12) — comparative interpretation with FDG and previous studies.
	23b	Discuss any limitations of the evidence included in the review.	Discussion §4 (pp. 11-14) — limitations of included studies (sample size, heterogeneity, lack of histopathology).
	23c	Discuss any limitations of the review processes used.	Discussion §4 (pp. 13-14) — limitations related to design, , and absence of bias meta-analysis.
	23d	Discuss implications of the results for practice, policy, and future research.	Discussion §4 (final paragraphs, pp. 14) — implications for diagnostic algorithms and multicenter trials.
**OTHER INFORMATION**			
Registration and protocol	24a	Provide registration information for the review, including register name and registration number, or state that the review was not registered.	Methods—PROSPERO (CRD420251238530) (p. 3).
	24b	Indicate where the review protocol can be accessed, or state that a protocol was not prepared.	Supplemetal table 1 (PRISMA checklist)
	24c	Describe and explain any amendments to information provided at registration or in the protocol.	Methods—PROSPERO (CRD420251238530) (p. 3).

Support	25	Describe sources of financial or non-financial support for the review, and the role of the funders or sponsors in the review.	Funding—no external funding (End Matter). p 15
Competing interests	26	Declare any competing interests of review authors.	Conflicts of Interest—none declared (End Matter).p 15
Availability of data, code and other materials	27	Report which of the following are publicly available and where they can be found: template data collection forms; data extracted from included studies; data used for all analyses; analytic code; any other materials used in the review.	Data Availability Statement—no new data; not applicable (End Matter). p 15

Table S2. Risk of bias.

Study (Year)	Confounding	Selection	Classification	Deviations	Missing Data	Outcome Measurement	Selective Reporting	Overall ROB	Why this overall judgment
**Yu 2006**	Serious	Moderate	Low	Moderate	Moderate	Moderate	Serious	**Serious**	Early single-arm phase IIa with multiple administration routes and limited outcome standardization.
**Pless 2004**	Serious	Serious	Low	Moderate	Serious	Moderate	Serious	**Serious**	Very small (n = 6) pilot; high uncertainty in follow-up and limited reporting.
**Sollini 2013**	Serious	Moderate	Low	Moderate	Serious	Moderate	Serious	**Serious**	Minimal PRRT subgroup, brief follow-up, substantial missing data, feasibility-focused design.
**Lapa 2016**	Serious	Moderate	Low	Low	Moderate	Moderate	Serious	**Serious**	Retrospective feasibility; acceptable reporting but major confounding and clinical selection.
**Assadi 2021**	Moderate	Moderate	Low	Moderate	Moderate	Moderate	Moderate	**Serious**	Phase I–II exploratory FAPI therapy with clear protocol but mixed tumors and variable doses.

**Serfling 2023**	Moderate	Moderate	Low	Low	Moderate	Moderate	Moderate	**Moderate**	Large retrospective imaging-impact study with reasonable completeness but no adjustment.
**Şen 2023**	Moderate	Moderate	Low	Low	Moderate	Moderate	Moderate	**Moderate**	Good reporting and standardized PET assessment; still observational without confounder control.
**Xie 2024**	Moderate	Moderate	Low	Low	Moderate	Moderate	Moderate	**Moderate**	Prospective, small sample, but standardized response assessment and acceptable follow-up.
**Fu 2025**	Moderate	Moderate	Low	Moderate	Moderate	Moderate	Moderate	**Moderate**	Prospective phase II with fixed dosing and strong methodological clarity; main issue = uncontrolled design.
**Liu 2025**	Moderate	Moderate	Low	Low	Moderate	Moderate	Moderate	**Moderate**	Well-reported first-in-human dose-escalation; consistent endpoints despite small cohorts.
